# Prolonged mechanical ventilation in a respiratory-care setting: a comparison of outcome between tracheostomized and translaryngeal intubated patients

**DOI:** 10.1186/cc8890

**Published:** 2010-03-01

**Authors:** Yao-Kuang Wu, Ying-Huang Tsai, Chou-Chin Lan, Chun-Yao Huang, Chih-Hsin Lee, Kuo-Chin Kao, Jui-Ying Fu

**Affiliations:** 1 Division of Pulmonary Medicine, Buddhist Tzu Chi General Hospital, No. 289, Jianguo Rd., Xindian City. Taipei, 231, Taiwan; 2 School of Medicine, Tzu Chi University, Hualien, No. 289, Jianguo Rd., Xindian City. Taipei, 231, Taiwan; 3 Division of Pulmonary and Critical Care Medicine, Chang Gung Memorial Hospital, No. 5 Fu-Shin Street, Gueishan, Taoyuan, 333, Taiwan; 4 Department of Respiratory Care, Chang Gang University, No. 5 Fu-Shin Street, Gueishan, Taoyuan, 333, Taiwan

## Abstract

**Introduction:**

Mechanical ventilation of patients may be accomplished by either translaryngeal intubation or tracheostomy. Although numerous intensive care unit (ICU) studies have compared various outcomes between the two techniques, no definitive consensus indicates that tracheostomy is superior. Comparable studies have not been performed in a respiratory care center (RCC) setting.

**Methods:**

This was a retrospective observational study of 985 tracheostomy and 227 translaryngeal intubated patients who received treatment in a 24-bed RCC between November 1999 and December 2005. Treatment and mortality outcomes were compared between tracheostomized and translaryngeal intubated patients, and the factors associated with positive outcomes in all patients were determined.

**Results:**

Duration of RCC (22 vs. 14 days) and total hospital stay (82 vs. 64 days) and total mechanical ventilation days (53 vs. 41 days) were significantly longer in tracheostomized patients (all *P *< 0.05). The rate of in-hospital mortality was significantly higher in the translaryngeal group (45% vs. 31%;*P *< 0.05). No significant differences were found in weaning success between the groups (both were >55%) or in RCC mortality. Because of significant baseline between-group heterogeneity, case-match analysis was performed. This analysis confirmed the whole cohort findings, except for the fact that a trend for in-hospital mortality was noted to be higher in the translaryngeal group (*P *= 0.08). Stepwise logistic regression revealed that patients with a lower median severity of disease (APACHE II score <18) who were properly nourished (albumin >2.5 g/dl) or had normal metabolism (BUN <40 mg/dl) were more likely to be successfully weaned and survive (all *P *< 0.05). Patients who were tracheostomized were also significantly more likely to survive (*P *< 0.05)

**Conclusions:**

These findings suggest that the type of mechanical ventilation does not appear to be an important determinant of weaning success in an RCC setting. Focused care administered by experienced providers may be more important for facilitating weaning success than the ventilation method used. However, our findings do suggest that tracheostomy may increase the likelihood of patient survival.

## Introduction

Increasingly frequently, patients maintained on prolonged mechanical ventilation (PMV) are given a tracheostomy [1]. Tracheostomy is thought to offer several advantages over traditional translaryngeal intubation, including improved physical and psychological comfort, decreased risk of inadvertent extubation, accelerated weaning from mechanical ventilation, decreased time of ICU stay before transfer to step-down facilities, and a reduced risk of developing ventilator-associated pneumonia [2,3]. Despite the increasing use of tracheostomy for PMV, currently no consensus exists as to whether this technique is associated with definite outcome benefits, as compared with translaryngeal intubation [4]. No study to date has compared the outcome of tracheostomy and translaryngeally intubated PMV patients in a specialized Respiratory Care Center (RCC) setting. All previous studies have been conducted in ICU settings.

The aim of the present study was to test the hypothesis that tracheostomy improves the outcome in patients maintained on PMV in an RCC setting. The major outcomes of interest were weaning success and mortality rate.

## Materials and methods

### Setting

Chang Gung Memorial Hospital is a 3,800-bed tertiary medical center containing 350 ICU beds. The 24-bed RCC unit was established in November 1999 as a part of a policy transferring responsibility for general ICU patients experiencing MV weaning difficulty.

### Patients and RCC admission criteria

All patients transferred to the RCC between November 1999 and December 2005 were identified. Patients were included in this study if they had been maintained on MV in excess of 3 weeks before RCC admission, and all previous weaning attempts had failed.

Patients were eligible for RCC admission if they met the National Health Insurance (Bureau of National Health Insurance, Taiwan) requirements: hemodynamic stability, no vasoactive drug infusion for 24 hours or more before transfer, stable oxygen requirements (fraction of inspired oxygen 40% or more, and positive end-expiratory pressure less than10 cm H_2_O), no acute hepatic or renal failure, no requirement for surgical intervention within the ensuing 2 weeks, or if the attending pulmonary physician deemed it beneficial for the patient to be transferred to the RCC. No other principal restrictions were placed on admission to the RCC. Admission decisions were not based strictly on diagnosis, route of MV, prognosis, weaning, or rehabilitation potential. Any patient who became hemodynamically unstable or had multiple organ failure was transferred back to the appropriate ICU. Most (97%) of the RCC-study patients were admitted from the institutional ICU. The remaining patients were transferred from other hospital ICUs.

Terminal cancer patients and those patients who had been given tracheostomies before RCC admission were excluded from this study. The reasons for excluding terminal cancer patients were short life expectancy and the fact that (in our experience) families of these patients tend to deny any request for tracheostomy. Although some patients were admitted to the RCC on more than one occasion during a single care episode, for statistical purposes, data were recorded for the first admission only.

Indications for tracheostomy included the following: necessity for PMV, failed extubation or reintubation, unrelieved upper-airway obstruction, airway protection (including the need of airway access to remove secretions), and avoidance of complications associated with translaryngeal intubation. All tracheostomies were performed by a surgeon or ear, nose, and throat specialist in a surgical operating room. Indications for continued translaryngeal intubation included a short predicated lifespan (less than 2 months) and refusal of tracheostomy by the patient or relative(s).

This study was approved by the Institutional Internal Review Board. Informed consent was obtained from either the patient or the patient's family at discharge.

### RCC description

Nurse-to-patient ratios in the RCC were 1:3, and respiratory therapist-to-patient ratios were 1:8. Specialists in pulmonary and critical care medicine served as primary physicians for all patients. In-hospital night coverage was provided by fellow trainees. Consultation services were available for most medical and surgical specialties.

The weaning process involved daily targets of either increasing periods of spontaneous breathing or a gradual reduction in pressure support. Other aspects of RCC care included identification of reversible causes of weaning failure, limited use of sedatives, restoration of normal sleep/wake cycles, attention to nutrition, pulmonary rehabilitation (including respiratory muscle training), and attempts to improve patient autonomy through methods such as establishing speech and self-feeding. Discharge planning was managed by nurse or social-work case managers. Hemodialysis was available in the RCC as required.

### Variables measured

The following variables were recorded for all study patients within 24 hours of admission: demographics, previous ICU type (medical or surgical; MICU or SICU), cause leading to PMV, duration of ICU and RCC stay, days on MV before RCC admission, total days on MV, day of tracheostomy after RCC admission (if the procedure was performed), Acute Physiology and Chronic Health Evaluation II (APACHE II) score, serum albumin, blood urea nitrogen (BUN) level, and blood gas data. "Total mechanical ventilation days" was defined as the time from initiation of MV to the time when weaning was successful or attempts were ceased. "Length of (hospital) stay" was defined as the time from ICU admission to the end of hospital care. The highest modified Glasgow Coma Scale scores (GCS: verbal score as one) were also obtained by nurses within the first 24 hours of admission. Rapid shallow breath indices (RSBIs), arterial oxygen pressure/fraction of inspiratory oxygen (PaO_2_/FIO_2_), and maximal inspiratory negative pressure (PI_max_) were also measured during spontaneous breathing. PI_max _values were determined as the mean of three measurements by using a Wright spirometer. PaO_2_/FIO_2 _was assessed within the first week of RCC admission. RCC and in-hospital mortality were calculated. RCC mortality was determined as the number of patients who died in the RCC divided by the total number of patients admitted to the RCC. In-hospital mortality was determined as the number of patients who died either at the RCC or before discharge, divided by the total number of patients admitted to the RCC. The numbers of comorbidities also were assessed [5,6]. These included the following: diabetes (as determined by history, or if admitted with diabetic ketoacidosis or hypovolemic hyperosmotic nonketotic coma, or if discharged on glucose-lowering medications); chronic obstructive or restrictive lung disease (as determined by history, radiographic imaging, or pulmonary function testing); congestive heart failure (significant systolic dysfunction as determined by echocardiography); coronary atherosclerotic disease; disabling neurologic conditions (including cerebrovascular accidents and neuromuscular disease); end-stage renal disease (requiring dialysis before admission); hepatic cirrhosis (as determined by abdominal echo); metastatic cancer; and acquired immunodeficiency syndrome.

PMV causes were classified into one of the following six categories [7]: acute lung injury (pneumonia, acute respiratory distress syndrome, aspiration injury, and chest trauma); chronic obstructive pulmonary disease (COPD); postoperative condition (coronary artery bypass grafting, abdominal surgery, or lobectomy); cardiac disease (acute myocardial infarction, congestive heart failure); neurologic disease (neuromuscular disease, cerebrovascular accident, cervical spinal injury, acquired critical neuromyopathy), or miscellaneous causes. Classifications were based on the reason that the patient could not be weaned, rather than the reason for MV initiation.

The number of patients successfully weaned and the length of time required for successful weaning were recorded. Patients were considered to be "ventilator independent" if mechanical ventilation was not required for 7 consecutive days and nights, regardless of outcome. Patients were considered to be "ventilator dependent" (including nocturnal mechanical ventilation) if weaning efforts were discontinued after both the interdisciplinary team and the informed patient/family agreed that these efforts should cease. No time limit was set for considering mechanical ventilation or weaning attempts. Patients who were classified as being ventilator dependent were transferred to a step-down respiratory-care ward for further long-term care.

### Statistical analysis

The two groups of patients were a tracheostomy group and a translaryngeal tube group. Comparisons were made between the tracheostomy and translaryngeal groups. Continuous data are expressed as mean ± standard deviation (SD) or median (range), whereas categoric data are expressed as frequencies and percentage. Baseline characteristics were compared with Student's *t *test, Wilcoxon rank-sum test (for skewed data), χ^2 ^test, or Fisher's Exact test, as necessary. Multivariate stepwise logistic regression models were used to assess factors associated with both successful weaning and survival in all patients (the factors entered into this analysis included gender, source of patient [from MICU/SICU], performing tracheostomy, reason for MV, ICU MV days, modified GCS score, APACHE II score, albumin, PI_max_, PaO_2_/FIO_2_, and BUN). Because of the significant baseline heterogeneity between the tracheostomy and translaryngeal groups (see Table 1); we further analyzed the data by performing a case-matched comparison. All demographic and clinical variables shown in Table 1 with *P *values < 0.25 were entered into multivariate analysis for predicting tracheostomy. Stepwise logistic regression was performed to remove covariates that had multivariable *P *values of > 0.25. Then, by using the coefficients of the final regression equation, a propensity score for undergoing tracheostomy was calculated for each patient. The predictors identified included APACHE II score, ICU MV days, PI_max_, and PaO_2_/FIO_2 _ratio. Thereafter, a case-matched comparison was performed by using statistical methods described in a previous publication [8]. In brief, this involved matching each tracheostomy patient with a single translaryngeal intubated patient who had a similar propensity score (within 0.1 on a scale from 0 to 1). When more than one matched patient was identified for a given case, the patient with the least number of missing laboratory-data values was selected as the matched patient. Matched analysis, mixed model, or general estimation equations were used for case match study comparisons. Several continuous variables were categorized by median value (albumin and BUN) or a clinically meaningful cut-off point (APACHE II score) for logistic regression. Data were analyzed by using SAS 9.0 statistical software (SAS Institute Inc., Cary, NC) and a value of *P *< 0.05 was considered statistically significant.

**Table 1:** Summary of demographic and clinical variables in the tracheostomy and translaryngeal tube groups

Variable	Tracheostomy (*n* = 985)	Translaryngeal tube (*n* = 227)	*P *value
Age (years) †	73.17 ± 15.04	73.56 ± 15.69	0.73
Male ‡	551 (55.94%)	107 (47.14%)	0.02*
Transferred from MICU ‡	691 (70.15%)	181 (79.74%)	< 0.01*
APACHE II†	18.58 ± 5.53	20.30 ± 5.86	< 0.01*
Reason for MV||			< 0.01*
Acute lung injury	229 (23.25%)	69 (30.40%)	
Chronic lung disease	207 (21.02%)	37 (16.30%)	
Postoperative	86 (8.73%)	21 (9.25%)	
Cardiac disease	145 (14.72%)	33 (14.54%)	
Neuronal	242 (24.57%)	37 (16.30%)	
Miscellaneous	76 (7.72%)	30 (13.22%)	
ICU MV (days)§	28 (0, 213)	27 (1, 123)	< 0.01*
Bedridden before admission‡	194 (19.70%)	52 (22.91%)	0.29
Sum of chronic comorbidities§	1 (0, 5)	1 (0, 4)	0.75
Sum of chronic comorbidities||			0.52
0	183 (18.58)	36 (15.86)	
1	404 (41.02)	104 (45.81)	
2	271 (27.51)	66 (29.07)	
3	111 (11.27)	18 (7.93)	
4	13 (1.32)	3 (1.32)	
5	3 (0.30)	0	
Required hemodialysis‡	157 (15.94%)	30 (13.22%)	0.31
Modified GCS†	8.91 ± 2.63	8.48 ± 2.94	0.04*
RSBI§	128 (7, 818)	122 (11, 550)	0.49
PI_max_§	27 (1, 75)	29 (8, 75)	0.02*
PaO_2_/FIO_2_§	251.5 (61, 700)	290 (105, 577)	< 0.01*
Albumin (g/dl)†	2.69 ± 0.49	2.64 ± 0.50	0.16
BUN (mg/dl)§	29 (3.3, 234)	30.5 (4.9, 296)	0.04*

Abbreviations: MICU, medical intensive care unit; APACHE II, Acute Physiology and Chronic Health Evaluation II; ICU, intensive care unit; MV, mechanical ventilation; RCC, respiratory care center; Glasgow Coma Scale; RSBI, rapid shallow breath indices; PI_max_, maximum inspiratory pressure at negative volume; PaO_2_/FiO_2_, arterial oxygen pressure/fraction of inspired oxygen; BUN, blood urea nitrogen. Data are presented as number (%); mean ± standard deviation; or median (range). *A statistically significant between-group difference (*P *< 0.05). † Compared with Student's *t *test. ‡ Compared with the χ^2 ^test. §Compared with the Wilcoxon rank-sum test. || Compared with Fisher's Exact test.

## Results

After excluding those patients who had been given a tracheostomy before RCC admission or who had terminal cancer, a total of 985 patients remained with tracheostomy and 227 patients with a translaryngeal tube included in the study. Table 1 summarizes the patient demographics and the clinical variables assessed with respect to MV grouping (tracheostomy or translaryngeal tube). Significant differences were found between gender distribution, origin of patients, APACHE II score, reason for MV, ICU MV days, modified GCS score, PI_max_, PaO_2_/FIO_2_, and BUN levels (*P *< 0.05 for all). Table 2 summarizes the outcome variables for the two groups. Significant between-group differences were found for all of the following: in-hospital mortality, length of hospital stay, RCC length of stay, and total mechanical ventilation days (all *P *< 0.01).

**Table 2:** Summary of outcome variables in the tracheostomy and translaryngeal tube groups

Variable	Tracheostomy (*n* = 985)	Translaryngeal tube (*n* = 227)	*P value*
Length of stay (days)§	82 (12, 806)	64 (1, 424)	< 0.01*
RCC length of stay (days)§	22 (0, 151)	14 (0, 151)	< 0.01*
Total MV days§	53 (8, 246)	41 (0, 216)	< 0.01*
Weaned†	549 (55.74%)	136 (59.91%)	0.25
In-hospital mortality†	303 (30.76%)	102 (44.93%)	< 0.01*
RCC mortality†	210 (69.31%)	69 (67.65%)	0.75

Abbreviations: MV, mechanical ventilation; RCC, respiratory care center. Data are presented as number (%). *A statistically significant between-group difference (*P *< 0.05). † Compared with Student's *t *test. §Compared with the Wilcoxon rank-sum test.

Table 3 shows the factors significantly associated with both successful weaning and survival in all patients, as determined using stepwise logistic regression. Patients who had a median severity of disease (APACHE II score <18), were adequately nourished (albumin >2.5), and had normal metabolism (BUN <40) were significantly more likely to be successfully weaned and to survive (*P *< 0.01 for all). Patients who had tracheostomies were borderline significantly more likely to be weaned (*P *= 0.06), and also significantly more likely to survive (*P *< 0.01).

**Table 3:** Factors associated with successful weaning and survival in mechanical ventilation patients as determined by using stepwise logistic regression

	Successful weaning		Survival	
	**OR (95% CI)**	***P *value**	**OR (95% CI)**	***P *value**

APACHE II <18	0.61 (0.48-0.79)	< 0.01*	0.61 (0.47-0.79)	< 0.01*
Albumin >2.5 g/dL	1.54 (1.22-1.95)	< 0.01*	1.80 (1.40-2.31)	< 0.01*
BUN <40 mg/dL	0.54 (0.42-0.70)	< 0.01*	0.45 (0.34-0.59)	< 0.01*
Performing tracheostomy	1.34 (0.99-1.82)	0.06	1.72 (1.26-2.34)	< 0.01*

Abbreviations: APACHE II, Acute Physiology and Chronic Health Evaluation II; BUN, blood urea nitrogen; OR, odds ratio. An APACHE II score >18 indicates more-severe disease. Albumin >2.5 indicates adequate nourishment. BUN >40 indicates abnormal metabolism. *A variable significantly associated with successful weaning or survival (*P *< 0.05).

As previously noted, because of the significant baseline heterogeneity between the tracheostomy and translaryngeal groups (see Table 1); we further analyzed the data by performing a case-matched comparison. Table 4 shows the results of this comparison. As expected, no significant between-group differences were found in any of the demographic or baseline variables assessed. No between-group differences were noted in weaning success or mortality between the groups. The length of stay, RCC length of stay, and the total number of mechanical ventilation days were significantly longer in the tracheostomy group (*P *= 0.04;*P *< 0.01; and *P *< 0.01, respectively).

**Table 4:** Case-matched study: summary of demographic and clinical variables in the tracheostomy and translaryngeal tube groups

	Tracheostomy (*n* = 129)	Translaryngeal tube (*n* = 129)	*P value*
Age (years)†	71.77 ± 17.30	74.43 ± 13.44	0.13
Male‡	62 (48.06%)	64 (49.61%)	0.80
Transfer from MICU‡	88 (68.22%)	94 (72.87%)	0.45
APACHE II†	20.02 ± 6.10	20.35 ± 5.50	0.58
Reason of MV‡			0.75
Acute lung injury	30(23.26)	28(21.71)	
Chronic lung disease	23(17.83)	18(13.95)	
Post op	11(8.53)	19(14.73)	
Cardiac disease	20(15.50)	23(17.83)	
Neuron	33(25.58)	23(17.83)	
Miscellaneous	12(9.30)	18(13.95)	
ICU MV (days)†	28 (9, 87)	27 (15, 65)	0.33
Bed ridden prior to admission‡	22 (17.05%)	29 (22.48%)	0.27
Sum of chronic comorbidities‡			0.70
0	31 (24.03)	27 (20.93)	
1	45 (34.88)	55 (42.64)	
2	37 (28.68)	37 (28.68)	
3	16 (12.40)	8 (6.20)	
4	0	2 (1.55)	
5	31 (24.03)	27 (20.93)	
Required hemodialysis‡	31 (24.03%)	19 (14.73%)	0.03*
Modified GCS†	8.67 ± 2.74	8.47 ± 2.96	0.52
RSBI†	148 (17, 418)	122 (11, 550)	0.36
PI_max_†	30 (1, 74)	29 (8, 75)	0.91
PaO_2_/FiO_2_†	289 (91, 700)	291 (112, 577)	0.35
Albumin (g/dL)†	2.80 ± 0.50	2.69 ± 0.42	0.06
BUN (mg/dL)†	31 (6, 215)	30 (4.9, 220)	0.95
Length of stay (days)§	84 (32, 806)	72 (19, 197)	0.04*
RCC length of stay (days)§	28 (5, 121)	16 (2, 151)	< 0.01*
Total MV days§	55 (21, 173)	44 (19, 186)	< 0.01*
Weaned||	84 (65.12%)	82 (63.57%)	0.79
In-hospital mortality||	19 (14.73%)	50 (38.76%)	0.08
RCC mortality||	12 (63.16%)	32 (64.00%)	0.72

Abbreviations: MICU, medical intensive care unit; APACHE II, Acute Physiology and Chronic Health Evaluation II; ICU, intensive care unit; MV, mechanical ventilation; RCC, respiratory care center; Glasgow Coma Scale; RSBI, rapid shallow breath indices; PI_max_, maximum inspiratory pressure at negative volume; PaO_2_/FiO_2_, arterial oxygen pressure/fraction of inspired oxygen; BUN, blood urea nitrogen. Data presented as: n (%); mean ± standard deviation; or median (range). *A statistically significant between-group difference (*P *< 0.05). † Compared with mixed model analysis. ‡ Compared with general estimation equation analysis. §Compared with mixed-model analysis, adjusted for the requirement of hemodialysis. || Compared with general estimation equation analysis, adjusted for the requirement hemodialysis.

## Discussion

Previous studies noted the need for specialized care units to manage respiratory rehabilitation [9]. Our study is the first to compare outcome between tracheostomized and translaryngeally intubated patients in a specialized regional weaning center for PMV. The fact that this investigation was undertaken in a specialized RCC reduced the influence of potential confounding factors, such as lack of staff experience and variability of setting, that are a problem in many studies.

Within our RCC, tracheostomy did not lead to increased weaning success as compared with translaryngeal intubation. Furthermore, our case-matched analysis revealed that no difference in either RCC or in-hospital mortality was present between the tracheostomy and translaryngeal tube-intubated patients. Multivariate analysis did reveal, however, that tracheostomy was a significant predictor of survival. Other studies have variously reported that tracheostomy is [8,10] and is not [9,11] associated with decreased ICU and in-hospital mortality rates. Further RCC studies are needed to confirm the findings regarding mortality and weaning success presented herein.

We also found that RCC and in-hospital lengths of stay and total MV days were significantly increased in tracheostomy compared with translaryngeally intubated patients. These findings are consistent with those of previous reports [8-10]. Whether decreased or increased length of stay is ultimately of benefit to the patient is dependent on the long-term results of treatment after leaving the hospital, something we did not measure.

Our study also reports specific biochemical markers that may be suitable indictors for identifying tracheostomy candidates. Specifically, we found that patients with BUN levels lower than 40 (indicating adequate metabolic functioning) and albumin concentrations greater than 2.5 (indicating adequate nutritional status) were significantly more likely to be successfully weaned and survive. On confirmation of these findings, assessment of the aforementioned markers may prove use in the clinical setting to facilitate the optimal management of PMV patients.

In this study, a significantly higher requirement for hemodialysis was found in the tracheostomy patients. Despite this, no corollary increase was found in the rate of mortality. This contrasts to the finding of Chao and colleagues [12], who reported that mortality was markedly increased in patients with concurrent PMV and renal-replacement therapy. A larger (although not significantly) number of patients in the tracheostomy group with end-stage renal disease required regular dialysis in our study (32.26% in the tracheostomy group and 26.32% in the translaryngeal tube-intubated patients). This may underlie the increased requirement for hemodialysis in this group of patients and explain the lack of an increase in mortality (patients in Chao's study had more severe renal dysfunction) [12].

Our study has a number of limitations that warrant mention. First, it should be noted that all tracheostomy patients received traditional surgical tracheostomies. Others have suggested that the popularity of the percutaneous tracheostomy technique is a major reason underlying the increased utilization of tracheostomy in PMV patients [8]. Hence in our analysis, we were not able to compare outcome with regard to tracheotomy technique (that is, percutaneous versus traditional surgical tracheostomy). 

Conversely, the homogeneity of our tracheostomy patient cohort in this respect could be viewed as a positive in terms of a decreased risk of technique-associated confounding. A further limitation is that we did not record data concerning decannulation of the tracheostomy, the effects of inadvertent extubation on the outcomes of the translaryngeally intubated group, tracheostomy complications, or the rate of ventilator-associated pneumonia in the different groups. Any of these factors could have influenced patient morbidity, mortality, or weaning ability.

Third, we did not assess the outcomes of patients after discharge. The long-term benefits (if any) of tracheostomy compared with translaryngeal intubation are yet to be determined.

Finally, our patients were not randomly assigned to the tracheostomy or translaryngeal-intubation groups. Although we used a case-matched method of statistical analysis, our data are confounded by the subjective decisions of the attending physicians to initiate tracheostomy. We also acknowledge that despite our best efforts to control for confounding factors, residual confounders associated with the different patient populations may have influenced our findings.

## Conclusions

Within a specialized respiratory care unit, successful weaning was not increased in tracheostomy compared with translaryngeally intubated patients. No between-group differences were found in RCC or in-hospital mortality, as determined by case-match analysis. Interestingly, tracheostomy was found to be a significant predictor of survival. These findings suggest that focused care administered by experienced providers, as occurs in a specialized care unit, is more important in facilitating weaning than is the ventilation method used. In our weaning and survival regression model, the subgroup of patients who exhibited the most-positive outcomes had lower BUN levels, higher albumin concentrations, moderate APACHE II scores, and tracheostomies. Given that tracheostomy was associated with increased survival, we suggest that this may be a better means of facilitating MV than is translaryngeal intubation.

## Key messages

• The type of prolonged mechanical ventilation does not appear to be an important determinant of successful weaning in a specialized respiratory care center.

• The subgroup of patients who fared best after mechanical ventilation had lower BUN levels, higher albumin concentrations, moderate APACHE II scores, and had tracheostomies.

• The significant association between tracheostomy and patient survival suggests that tracheostomy may be the optimal method of mechanical ventilation.

## Abbreviations

APACHE: Acute Physiology and Chronic Health Evaluation; BUN: blood urea nitrogen; COPD: chronic obstructive pulmonary disease; GCS: Glasgow Coma Scale; ICU: intensive care unit; MICU: medical intensive care unit; PaO_2_/FIO_2_: arterial oxygen pressure/fraction of inspiratory oxygen; PI_max_: maximal inspiratory negative pressure; PMV: prolonged mechanical ventilation; RCC: respiratory care center; RSBI: rapid shallow breath index; SD: standard deviation; SICU: surgical intensive care unit.

## Competing interests

The authors declare that they have no competing interests.

## Authors' contributions

YKW contributed to the study design, data processing, and drafting of the manuscript. CYH, CHL, and KCK participated in data collation.
